# The role of emotion and reflection in the development of student teachers’ self-efficacy when analyzing video lessons

**DOI:** 10.3389/fpsyg.2023.1080883

**Published:** 2023-02-03

**Authors:** Anne Schlosser, Jennifer Paetsch

**Affiliations:** Institute of Educational Science, University of Bamberg, Bamberg, Germany

**Keywords:** self-efficacy, emotion, emotional arousal, reflection, teacher education, video lessons

## Abstract

**Introduction:**

Teachers’ self-efficacy is an important indicator of student teachers’ preparedness for teaching. Interventions using video lessons are effective in increasing student teachers’ self-efficacy. However, there is a lack of research on emotional and reflective processes in the context of video-based interventions.

**Methods:**

The present study examined emotions and reflection as well as their effects on changes in self-efficacy in a video-based intervention. A total of 159 student teachers participated in the study. The participants were randomly assigned to three groups: Two groups analyzed video lessons in which group roup one received open-ended observation tasks (ig1) and group two received structured observation tasks (ig2). Participants in the control group (cg) analyzed text-based case studies with open-ended observation tasks.

**Results:**

The results show that self-efficacy increased with medium effect size (*d* = 0.68) in video group two (ig2), whose members analyzed videos using structured observation tasks but not in video group one (ig1), whose members analyzed open-ended observation tasks, and in the control group. In addition, there were significant relations between positive arousal and reflection. Finally, regression analyses showed that reflection was a significant predictor for changes in self-efficacy, whereas no significant effect of emotional arousal was detected.

**Discussion:**

In conclusion, the findings of this study indicate that video-based interventions with structured observation tasks increased self-efficacy among student teachers. Furthermore, the findings provide novel evidence on the association between reflection, self-efficacy and emotion in video-based interventions in teacher education.

## 1. Introduction

In previous empirical studies, self-efficacy turned out to be a relevant factor influencing pre-service and in-service teachers’ acquisition of competencies, professional teacher behavior (e.g., Klassen et al., 2011), and teacher health (e.g., Evers et al., 2002; Friedman, 2003; Hoy and Spero, 2005). Thus, it can be inferred that supporting the development of self-efficacy is an appropriate goal in university teacher training (e.g., Enochs et al., 2000; Gaudin and Chaliès, 2015). Moreover, it is already known that student teachers’ self-efficacy can be increased in different learning environments. For example, there is evidence that collaborative learning environments with theoretical input can increase self-efficacy (e.g., Valtonen et al., 2015), and interventions with practical content (e.g., microteaching, counseling training; e.g., Hertel, 2009; Mergler and Tangen, 2010) or teaching practicums (e.g., Pfitzner-Eden, 2015) are also effective.

Video-based interventions seem to be effective in promoting self-efficacy (e.g., Thiel et al., 2020). Learning with video lessons can be characterized as a vicarious learning experience and has become a frequently used method for linking theory and practice in teacher education (Bandura, 1986; Gaudin and Chaliès, 2015). Video lessons allow students to observe complex teaching situations without a requirement to act immediately. Moreover, the flexible pausing and playing of the video allow for situation-specific incorporation of different theoretical perspectives (e.g., Sherin, 2004). Thus, video lessons enable students to approach professional teacher action known as “approximations of practice” (Grossmann et al., 2009) and offer learners time and space for an analysis and critical reflection without pressure to act. In addition to the development of self-efficacy, empirical studies show that through video lesson interventions, action-oriented knowledge can be acquired and professional vision can be trained (e.g., Sherin and van Es, 2009).

Whereas some studies show that video-based interventions trigger reflective processes (e.g., Seidel et al., 2011; Zhang et al., 2011), few studies focus on examining the emotional-motivational aspects in video-based interventions. Empirical studies found that student teachers experience more positive emotions (e.g., Syring et al., 2015; Egloff and Souvignier, 2020) and higher emotional arousal (e.g., Egloff and Souvignier, 2020) while working with video lessons compared to student teachers working with text-based lessons, in control groups. However, the role of reflection and emotions in professional development in video-based learning environments remains unclear.

To address this research gap, the present study focused on beginning student teachers’ self-efficacy concerning adaptive teaching in heterogeneous classrooms and their changes during a video-based intervention. The video lessons showed typical teaching situations for dealing with heterogeneity in elementary schools, with a focus on the different performance levels and requirements for students. Our study explored the emotional and reflective processes among student teachers analyzing video lessons or equivalent written case studies and investigated the effects of emotion and reflection on changes in their self-efficacy.

## 2. Theoretical framework

### 2.1. Development of self-efficacy in video-based interventions

According to Bandura (1986), individuals shape self-efficacy by interpreting information regarding their abilities. This information stems from four sources: personal experience, vicarious experience, verbal persuasion, and affective and physiological states.

Personal experience is the most effective source because it provides information about one’s successes or failures. Self-efficacy is positively influenced in the case of success and negatively in the case of failure. In vicarious experiences, learning occurs from models. Individual self-efficacy is affected by observing model competency behaviors and comparing them to one’s competencies. Vicarious experiences are particularly effective when different successful models can be compared. Verbal persuasion of meaningful others can have an impact on one’s self-efficacy. Finally, physiological and affective states provide information about arousal during situations in which capabilities are practiced. In particular, negatively evaluated somatic information decreases self-efficacy, and positively read somatic information increases self-efficacy (*cf.*, Bandura, 1986).

A few empirical studies revealed associations between emotions and teacher self-efficacy (e.g., Stephanou et al., 2013; Burić et al., 2020). However, research on the sources of self-efficacy in teacher education is scarce (e.g., Klassen et al., 2011; Pfitzner-Eden, 2015; Keppens et al., 2021). The present study addresses this research gap by examining the effects of emotions and reflection in a vicarious learning experience (analyzing video lessons), for changes in self-efficacy.

Video lessons have become a popular tool to foster teacher self-efficacy (e.g., Thiel et al., 2020). Various types of videos can be used, such as videos of one’s teaching, videos of peers, typical video lessons, and scripted videos (these are videos made with actors; e.g., Blomberg et al., 2013). For example, results of an intervention study show that self-efficacy increases more in students who analyzed videos of others or in students who analyzed videos of others and their own videos than in a group in which students analyzed text-based case studies and their own teaching protocols. In sum, the superiority of video-based interventions compared to text-based interventions is evident though it does not seem relevant if one’s own or another’s video is analyzed (Gold et al., 2017). However, in addition to analyzing authentic videos, it is also possible to use scripted videos, such as the best and worst examples of teaching practices (Blomberg et al., 2013). In their intervention study, Thiel et al. (2020) investigated whether functional or dysfunctional scripted video scenarios had different effects on student teachers’ professional development. The results showed significant increases in self-efficacy according to student engagement, classroom management, and instruction in both experimental groups.

### 2.2. Emotions in video-based interventions

Emotional experience is described as a complex phenomenon that arises, is maintained, and diminishes in reciprocal dependence on cognitions. Furthermore, emotions are integrated into current action and interact with goals, expectations, and subjective evaluations (Lazarus and Lazarus, 1994). Emotions can be categorized into valence (pleasant-unpleasant) and arousal (low-high; e.g., Feldman Barrett and Russell, 1998). Thus, arousal arises after the attribution of meaning, which is affected by the assessment of one’s control and subjective values (e.g., Lazarus and Lazarus, 1994; Pekrun, 2006). In addition, emotional experience depends on individual (e.g., gender) and contextual factors (e.g., social, geographical, political; e.g., Boler, 1997; Nussbaum, 2001; Feldman Barrett et al., 2007). The experience of emotions is, relevant to shaping learning processes as well as one’s teacher’s actions (Boler, 1997).

In addition to activating cognitive and motivational processes, video lessons are likely to engage student teachers emotionally as well (Gaudin and Chaliès, 2015). Since videos provide a more authentic learning experience about a teaching situation compared to text-based case studies, it can be assumed that they provide more emotional involvement than text-based case studies. Few studies refer to the emotional aspects in the context of analyzing video lessons (e.g., Syring et al., 2015; Egloff and Souvignier, 2020). Egloff and Souvignier (2020) examined the emotional experience (arousal and valence) of students who analyzed video lessons and students who analyzed videos of expert talks (control group). Both videos addressed student-oriented teaching beliefs, attitudes, and intentions positively. The results showed a significantly higher emotional arousal in students who analyzed video lessons than for participants of the control group. In addition, higher emotional arousal was associated with a more positive change in student teachers´ beliefs, whereas emotional valence did not affect changes in teaching-related beliefs, attitudes, and intentions. The authors attribute the results to the fact that video lessons are authentic situations that elicit greater emotional responses (*cf.*, Egloff and Souvignier, 2020). Overall, it has been shown that learning with video lessons is emotionally involved and can generate emotional arousal.

### 2.3. Reflection in video-based interventions

Another strand of theory addresses the role of reflection in authentic learning situations in (student) teachers’ professional development (*cf.*, Gaudin and Chaliès, 2015). Although there is widespread agreement that reflection is crucial to improving teacher practice, there is, at the same time, a lack of clarity about different definitions of reflection (for an overview see Clara, 2015). Based on Schön’s (1987) and Dewey’s (1910) writings, reflection is a complex construct that can be described as a specific and purposeful thought process. Reflection seems particularly relevant for the professionalization of pre-service teachers as it can stimulate theory-practice connections and critical thinking (*cf.*, Bain et al., 1999; Mulryan-Kyne, 2020). Therefore, reflection has an important role in teacher education and is crucial for linking theory and practice as well as for the development of competencies (e.g., Clara, 2015). Reflection in classroom situations is difficult to implement as it is dynamic and complex. Therefore it seems more appropriate and more in line with the goal of teacher education to reflect on events after some time has passed (e.g., Anderson, 2019; Neuweg, 2021). In conclusion, reflective processes should lead to an understanding of actions, deeper observations, and future drive for action (e.g., Dewey, 1910; Zeichner, 1981).

Reflection in the context of video lesson analysis describes comprehensive reflection on teaching practice while viewing a video and on one’s own beliefs. Elsner et al. (2020) developed a model of the reflection processes for video lessons that include the following components: (1) observation of the teaching situation, (2) recognition of relevant scenes, (3) representation (description) of the recognition scene, (4) theory-based reflection of the scene, and (5) development of alternative scenarios (Elsner et al., 2020). Reflection on teaching situations can be done in an open or highly structured way. In general, it is known that novices have a more shallow reflective competence and need more guidance compared to experts (e.g., Blomberg et al., 2013; Gaudin and Chaliès, 2015). Structured observation tasks are especially advantageous for students with little previous knowledge because they guide students’ focus on critical events in video lessons, which can be helpful for deeper reflection. In addition, structured observation tasks can also help to appropriately reduce excessive cognitive load in complex learning situations (e.g., Blomberg et al., 2013; Elsner et al., 2020). Thus, it may be important to guide students in a structured way in their analysis to gain a greater depth of reflection (e.g., Kumschick et al., 2017; Imhof and Schlag, 2018; Elsner et al., 2020).

According to Bandura (1986), there is an association between reflection and self-efficacy since mastery or vicarious experiences must be interpreted by the individual to impact one’s self-efficacy. Moreover, Tschannen-Moran et al. (1998), who describe in their model the theoretical underpinnings of teacher self-efficacy, propose that reflective processes, such as the self-assessment of one’s own competencies, affect changes in teacher self-efficacy. Associations between reflection and self-efficacy have been shown in empirical studies (e.g., Han and Wang, 2021; Naidoo and Naidoo, 2021), but not in the context of video lessons.

In the context of learning with video lessons, it is particularly interesting how reflection and emotions interact. Several theories describe the relationship between emotion and cognitive processes (e.g., Frederickson, 2001; Um et al., 2012). For example, the emotions-as-facilitators-of-learning hypothesis describes a favoring of the learning process through the positive influence of emotions on cognitive processing (*cf.* Um et al., 2012). This is shown in different empirical studies (e.g., Isen and Baron, 1991; Erez and Isen, 2002; Konradt et al., 2003). A qualitatively designed case comparison with in-service teachers points to the association of depth of reflection and emotional experience while analyzing videos (Kleinknecht and Poschinski, 2014), but quantitative research findings in this context are still lacking.

## 3. Research question and hypotheses

The relevance of using video lessons to promote professional competencies in teacher education was emphasized (e.g., Borko et al., 2011). Video lessons are vicarious experiences and a source of self-efficacy, according to Bandura’s (1986) self-efficacy theory. It has been shown, empirically, that analyzing video lessons can increase self-efficacy (e.g., Gold et al., 2017; Thiel et al., 2020). Emotional states are another source of self-efficacy (Bandura, 1986) and the results of empirical studies show that emotion and self-efficacy are associated with each other (e.g., Pitkäniemi, 2017). In addition, empirical studies show that the analysis of video lessons stimulate emotional experience (e.g., Kleinknecht and Poschinski, 2014; Egloff and Souvignier, 2020).

Furthermore, analyzing video lessons encourages students´ reflection skills (e.g., Blomberg et al., 2013). Emotion and reflection have been investigated independently in the context of analyzing video lessons (e.g., Zhang et al., 2011; Egloff and Souvignier, 2020). Concerning reflection, there are few studies that have examined its relationship to self-efficacy, and research on reflection processes and emotional-motivational processes is still pending (e.g., Han and Wang, 2021). In video lesson research, one qualitative study of in-service teachers has highlighted that emotional experience is associated with depth of reflection (Kleinknecht and Poschinski, 2014). A few studies show that reflection and self-efficacy are associated with each other (e.g., Han and Wang, 2021; Naidoo and Naidoo, 2021), but not in the context of video lesson research. The question of the exact relationship between reflection, emotion, and self-efficacy in the context of video lessons remains open.

In our study, a 90-min intervention was administered to student teachers in a pre-post-experimental design. Students analyzed two video lessons (ig), or two equivalent written case studies (cg). Participants were asked to observe the case studies using either open-ended (ig1, cg) or structured observation tasks (ig2).

Against the theoretical and empirical background of the subject area, the present study examined the following questions:

*RQ 1:* Does student teachers’ self-efficacy increase during a video-based intervention?

*RQ 2:* What role do emotion and reflection play in changes in self-efficacy during a video-based intervention?

To answer the research questions, we developed four hypotheses. First, we assumed that self-efficacy increases while analyzing video lessons (e.g., Thiel et al., 2020). Therefore, we hypothesized that self-efficacy will increase more in ig2 (analyzing video lessons, structured observation tasks) than in ig1 (analyzing video lessons, open-ended observation tasks), and cg (analyzing text-based cases, open-ended observation tasks)–ig2 > ig1 > cg (*hypothesis 1*).

Second, Banduras’ self-efficacy theory (1986) allows for the assumption that analyzing video lessons is more stimulating for students’ emotional arousal than reading case studies. Empirical findings verify this assumption (e.g., Miesera and Will, 2017; Egloff and Souvignier, 2020). We hypothesized that participants of both intervention groups (ig1 and ig2) will experience more emotional arousal than participants in cg (*hypothesis 2a*). In addition, due to the variation of the observation tasks, different depths of reflection can be expected with the highest values on reflection in structured observation tasks. Therefore, participants in ig2 will report a deeper reflection than participants in ig1 and cg (*hypothesis 2b*).

Third, theoretically and empirically, there is evidence for a correlation between reflection and emotion (e.g., Kleinknecht and Poschinski, 2014; Stark, 2016). Consequently, we assume that emotion and reflection are associated with each other (*hypothesis 3*).

Finally, there is evidence for bilateral correlations between reflection and self-efficacy (e.g., Naidoo and Naidoo, 2021) as well as for emotion and self-efficacy (e.g., Pitkäniemi, 2017). Therefore, a theory-based (Bandura, 1986) hypothesis can be made that (a) emotion and (b) reflection will predict changes in self-efficacy in both intervention groups while controlling for previous experience in analyzing video lessons, interest and relevance, and reflection skills (*hypothesis 4*).

## 4. Methods

### 4.1. Intervention and procedures

The intervention was implemented on elementary student teachers in an online learning environment. Data were collected during a 90-min intervention in November 2021. After a short welcome in the plenum, in which the students received information about the learning unit and the link to a virtual campus, they were assigned randomly into three groups. Each group received a digital schedule *via* the virtual campus and filled in the first questionnaire (pretest). Then they received theoretical input on teaching in heterogeneous classes (e.g., definition, empirical results, and best practice) in an online learning environment. After completing the first case study,^1^ they filled in another questionnaire (posttest 1); Following the second case study,^2^ they completed the last questionnaire (posttest 2), which ended the seminar session for the students. Depending on intervention groups (ig) or control group (cg), the case studies were either video lessons that the students watched *via* the Metavideoportal^3^ or text-based case studies, which were the transcripts of the video lessons in edited form. Both videos showed examples of similar situations—task introductions in elementary school showing typical practice. Both positive and negative aspects on the topic of heterogeneity were observable in each case. Furthermore, the observation tasks varied within the groups: ig1 and the cg received two open-ended tasks (e.g., What did you notice positively in the video sequence?), ig2 received nine structured observation tasks (e.g., Describe the procedure for grouping! What do you think about this approach concerning teaching in heterogeneous classes? Give brief reasons for your answer!). The structured observation tasks were developed using Elsner’s et al. (2020) model, including description of relevant scenes, theory-based reflection, and the development of alternatives. Observation tasks were given to the students before they watched the video or read the text, the editing should be done after watching the video.

### 4.2. Sample

Participants were drawn from students enrolled at an elementary teacher education lecture and seminars at a German University. The University of Bamberg is a public university in Bavaria with a high percentage of teaching students (about 20%). Participants of our study were intended to work in elementary schools and their studies focused on primary education. The initial sample was *N =* 156, but with a relatively high dropout of 39% (*N* = 62) during the study. However, this was not considered a systematic dropout. There were no significant differences between participants who participated fully and those who dropped out early during the study (*p* = 0.094) or with regard to prior experience with the topic heterogeneity (*p* = 0.413), prior experience with video lesson analysis (*p* = 0.478), self-efficacy (*p* = 0.591), and positive (*p* = 0.267) and negative emotional arousal (*p* = 0.477). The participants were randomly assigned to three groups (according to their birth month), resulting in an initial *N* = 58 in ig1, *N* = 47 in ig2 and *N* = 51 in cg. A total of 87.7% of the participants were female, 10.3% are male, and 10 participants did not answer the question on gender. In all three groups, students were, on average, in their 2nd semester (*M* = 1.45, SD = 1.83). Participants in both intervention groups were, on average, 20 years old (ig1: *M* = 20.80, SD = 2.74; ig2: *M* = 20.62; SD = 2.98). Participants in the cg were, on average, 21 years old (*M* = 21.20, SD = 3.48).

### 4.3. Instruments

To investigate the hypotheses we used three measurement tools to evaluate self-efficacy, emotion, and reflection. Furthermore, previous experience, reflection skills, and interest and relevance were used as control variables.

#### 4.3.1. Self-efficacy

The *Self-efficacy of Adaptive Teaching in Heterogeneous Classrooms* by Meschede and Hardy (2020) was used to assess self-efficacy in pretest and posttest 2. The focus on teaching achievement in heterogeneous classes was in line with theoretical input, case studies, and the observation tasks of this intervention. The scale consisted of eight items. Participants had to rate themselves on a 4-point Likert scale (e.g., *I feel able to make reasoned decisions about differentiation in the classroom.*). The reliability of this scale was good to very good (see Table 1).

**Table 1:** Reliability coefficients.

	Pretest		Posttest 1		Posttest 2	
	*α*	*n*	*α*	*n*	*α*	*n*
Positive arousal	0.86	136	0.89	91	0.91	82
Negative arousal	0.83	137	0.85	92	0.89	82
Self-efficacy	0.81	129	-	-	0.84	76
Reflection (theoretical contextualization)	-	-	0.66	93	0.72	79
Reflection (theoretical evaluation)	-	-	0.86	92	0.91	80
Reflection skills	0.76	136	-	-	-	-
Interest and relevance	-	-	-	-	0.69	81

#### 4.3.2. Emotion

Emotional arousal was assessed with the PANAS (Positive and negative affect schedule; Breyer and Bluemke, 2016). Participants had to rate 20 adjectives on a 5-point Likert scale (e.g., *active, strong, irritated*). However, 10 adjectives belonged to positive arousal on the scale, and 10 items to negative arousal. The higher the mean values, the higher the arousal. The internal consistencies were good to very good (see Table 1).

#### 4.3.3. Reflection

To evaluate the depths of reflection, two subscales of the reflection circle by Reinders (2016) were used. The scales were about *theoretical evaluation* (including seven items; e.g., *Theories help me to better understand educational situations that I have experienced*.) and *theoretical contextualization* (including eight items; e.g., *I understand well how a theoretical concept can describe the situation.*). Participants had to rate their answers on a 4-point-Likert scale. The reliability of these scales was good to very good (see Table 1).

#### 4.3.4. Control variables

Participants had to rate their general reflection skills (self-developed scale including four items; e.g., *I think a lot about my role as a teacher in heterogeneous classes.*) on a 7-point Likert scale. A categorical variable was used to investigate previous experience (e.g., *Do you have previous experience with analyzing video lessons?*). Additionally, interest and relevance were also rated on a 4-point Likert scale (5 items, e.g., *The seminar session today fostered my interest in the topic area of Teaching in Performance Heterogeneous Classes;* adapted Reinders, 2016) and measured last (posttest 2). The reliability of the scales measuring reflection skills and interest and relevance was good (see Table 1).

### 4.4. Statistical analysis

The statistical software package Social Science (SPSS), Version 23, was used for the statistical analysis of the data. To identify changes in self-efficacy, a two-factor repeated measures ANOVA and single ANOVAs with repeated measurement for each group to examine group differences were conducted (*hypothesis 1*). We checked the differences for emotional arousal as well as for reflection using two-factor ANOVAs with repeated measures and planned contrast comparisons (*hypothesis 2*). The planned contrasts were adjusted to the hypotheses: In hypothesis 2a, the video groups (ig1 and ig2) were compared with the control group. In hypothesis 2b, the group with structured observation tasks (ig2) was compared with the two groups with open-ended observation tasks. Correlation analyzes were conducted to evaluate the association between emotional arousal and reflection (*hypothesis 3*). Finally, the prediction of self-efficacy was checked with a regression analysis. Self-efficacy (posttest 2) was the dependent variable, and emotion and reflection were the independent variables. Self-efficacy (pretest), previous experience in analyzing video lessons, general reflection skills, interest, and relevance were the control variables in the regression models. To control for multicollinearity, stepwise sequential regression models were calculated (*hypothesis 4*). For all parametric tests, Cohen’s *d* was calculated and interpreted according to Cohen’s (1988) benchmarks, as follows: ≥ 0.02 *small effect*, ≥ 0.05 *medium effect*, and ≥ 0.08 *large effect*.

## 5. Results

### 5.1. Descriptive results

All means for *positive emotional arousal* were in the middle range, with the highest values observed in ig2 (*M*_pretest_ = 3.00, SD = 0.77; *M*_posttest1_ = 3.12, SD = 0.82; *M*_posttest2_ = 2.85, SD = 0.96). Descriptively, cg showed a higher mean in posttest 1 (*M*_posttest1_ = 3.02, *SD* = 0.71) compared to the means of the other two measurement points (*M*_pretest_ = 2.82, SD = 0.60; *M*_posttest2_ = 2.71, SD = 0.80). The means for positive emotional arousal in ig1 were constant (see Table 2). The standard deviation increases slightly in all groups (see Table 2). However, the means for *negative emotional arousal* were in the lower range of the scale across all groups with only a few differences. In ig1, (*M*_pretest_ = 1.48, SD = 0.49; *M*_posttest1_ = 1.40, SD = 0.52; *M*_posttest2_ = 1.38, SD = 0.58) and ig2 (*M*_pretest_ = 1.45, SD = 0.52; *M*_posttest1_ = 1.32, SD = 0.44; *M*_posttest2_ = 1.21, SD = 0.25), negative emotional arousal decreased slightly during the intervention, and the mean value in cg increased again at posttest 2 (*M*_posttest2_ = 1.38, SD = 0.53).

**Table 2:** Descriptive statistics.

	Pretest				Posttest 1				Posttest 2			
	*M*	SD	*N*	N_mis_	*M*	SD	*N*	N_mis_	*M*	SD	*N*	N_mis_
Intervention group 1
Positive emotional arousal^a^	2.94	0.69	53	4	2.98	0.71	39	18	2.92	0.84	39	18
Negative emotional arousal^a^	1.48	0.49	52	5	1.40	0.52	39	18	1.38	0.58	39	18
Self-efficacy^b^	2.95	0.40	52	5	-	-	-	-	2.95	0.48	34	23
Reflection (theoretical contextualization)^b^	-	-	-	-	2.36	0.47	40	17	2.37	0.55	35	22
Reflection (theoretical evaluation)^b^	-	-	-	-	2.77	0.65	40	17	2.82	0.68	36	21
General reflection skills^c^	5.08	1.07	52	5	-	-	-	-	-	-	-	-
Interest and relevance^b^	-	-	-	-	-	-	-	-	3.98	0.89	35	22
Intervention group 2
Positive emotional arousal	3.00	0.77	39	5	3.12	0.82	21	23	2.85	0.96	18	26
Negative emotional arousal	1.45	0.52	41	3	1.32	0.44	22	22	1.21	0.25	18	26
Self-efficacy	2.96	0.40	37	7	-	-	-	-	3.08	0.34	18	26
Reflection (theoretical contextualization)	-	-	-	-	2.60	0.46	22	22	2.67	0.48	18	26
Reflection (theoretical evaluation)	-	-	-	-	3.05	0.47	22	22	2.85	0.80	18	26
General reflection skills	4.83	0.97	37	7	-	-	-	-	-	-	-	-
Interest and relevance	-	-	-	-	-	-	-	-	4.13	0.61	18	26
Control group
Positive emotional arousal	2.82	0.60	44	2	3.02	0.71	31	15	2.71	0.80	25	21
Negative emotional arousal	1.35	0.38	44	2	1.28	0.38	31	15	1.38	0.53	25	21
Self-efficacy	2.85	0.52	40	6	-	-	-	-	2.85	0.42	24	22
Reflection (theoretical contextualization)	-	-	-	-	2.50	0.55	31	15	2.49	0.55	26	20
Reflection (theoretical evaluation)	-	-	-	-	2.82	0.46	30	16	2.80	0.41	26	20
General reflection skills	5.03	1.15	42	4	-	-	-	-	-	-	-	-
Interest and relevance	-	-	-	-	-	-	-	-	3.82	0.68	24	22

^a^Ratings on a 5-point likert scale. ^b^Ratings on a 4-point Likert scale. ^c^Ratings on a 7-point Likert scale.

There were no differences in *self-efficacy* between pretest and posttest 2 in ig1 (*M*_pretest_ = 2.95, SD = 0.40; *M*_posttest2_ = 2.95, SD = 0.48) and cg (*M*_pretest_ = 2.85, SD = 0.52; *M*_posttest2_ = 2.85, SD = 0.42). In ig2 (*M*_pretest_ = 2.96, SD = 0.40; *M*_posttest2_ = 3.08, SD = 0.34) there was a slight, observable increase. All mean values were in the upper range of the 4-point Likert scale.

Concerning *reflection*, means for *theoretical contextualization* were in the middle range. The lowest means were observed in ig1 (*M*_posttest1_ = 2.36, SD = 0.47; *M*_posttest2_ = 2.37, SD = 0.55), followed by the mean values of cg (*M*_posttest1_ = 2.50, SD = 0.55; *M*_posttest2_ = 2.49, SD = 0.55), and finally, the highest mean values were in ig2 (*M*_posttest1_ = 2.60, SD = 0.46; *M*_posttest2_ = 2.67, SD = 0.48). The means of *theoretical evaluation* were, in general, higher than those of *theoretical contextualization*. Moreover, a similar picture emerges in all groups as in the scale theoretical evaluation. The lowest mean values were observable in ig1 (*M*_posttest1_ = 2.77, SD = 0.65; *M*_posttest2_ = 2.82, SD = 0.68). This was followed by the mean values of cg (*M*_posttest1_ = 2.82, SD = 0.46; *M*_posttest2_ = 2.80, SD = 0.410), and finally, the highest mean values were in ig2 (*M*_posttest1_ = 3.05, SD = 0.47; *M*_posttest2_ = 2.85, SD = 0.80).

The *control variables* were collected at one measurement point. First, reflection skills (pretest) showed high means, with the highest values in ig1 (*M* = 5.08, SD = 1.07) and cg (*M* = 5.03, SD = 1.15), compared to ig2 (*M* = 4.83, SD = 0.97). There were high standard deviations in all groups. Second, 42.1% in ig1, 54.4% in ig2, and 56.5% in cg indicated students’ previous experience in analyzing video lessons (pretest). Third, interest and relevance (posttest) showed the highest mean values in ig2 (*M* = 4.13, SD = 0.61), compared to cg (*M* = 3.82, SD = 0.68) and ig1 (*M* = 3.98, SD = 0.89). All mean values were in the higher range of the 5-point Likert scale.

### 5.2. Changes in self-efficacy

**Figure 1:**
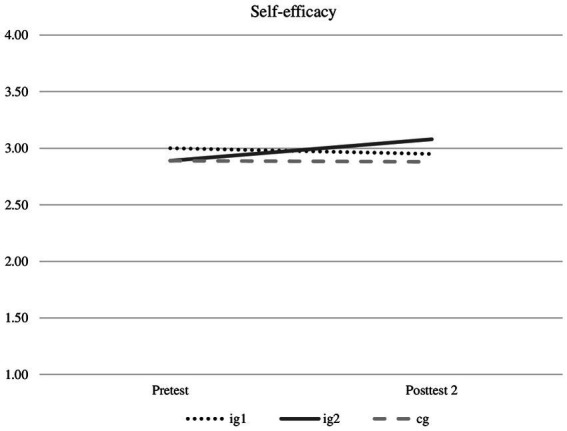
N_pretest_=72, N_posttest_=72.

In hypothesis 1 it was assumed that self-efficacy increases most in ig2, compared to ig1 and also to cg (ig2 > ig1 > cg). The calculation of a two-factor repeated measures ANOVA (*N* = 72) shows no significant overall improvement in self-efficacy (*p* = 0.228), but a significant interaction effect between measurement repetition factor and grouping factor [*F*(2, 69) = 4.40, *p* = 0.016, *ŋ*^2^ = 0.113] with a medium effect size (*d* = 0.71). The calculation of single ANOVAs with repeated measures for the different groups showed that differences between Pretest and Posttest 2 were significant only in ig2 [*F*(1,17) = 7.84, *p* = 0.012, partial ŋ^2^ = 0.316] and showed a medium effect (*d* = 0.680). Even after the Bonferroni correction, the effect was significant. In ig1 (*p* = 0.253) and cg (*p* = 0.871) there are no significant changes (see Figure 1). In sum, changes in self-efficacy were only apparent in ig2, whereas no significant changes were found in the other two groups. Therefore, hypothesis 1 may be partially accepted.

### 5.3. Emotion and reflection

It was assumed that participants in both intervention groups (ig1 and ig2) experienced more positive and negative emotional arousal than participants in cg. The two-factor ANOVAs with repeated measures show a significant overall improvement in positive emotional arousal [*F*(1.60,118) = 4.68, *p* = 0.017, *d* = 0.51], but no significant interaction of emotion and the group factor [*F*(3.19, 118) = 0.61, *p* = 0.621, *d* = 0.29]. There was no significant main effect [*F*(1.75, 132.9) = 1.06, *p* = 0.342, *d* = 0.24] and no interaction effect [*F*(3.50, 132.9) = 1.13, *p* = 0.342, *d* = 0.35] for negative emotional arousal (see Table 3). Nevertheless, hypothesis 2a must be rejected.

**Table 3:** Differences in emotional arousal and reflection (two-factor ANOVA with repeated measures).

		*F*	df	*p*	*d*
Positive arousal^a^	Main effect	4.68	1.60	0.017	0.51
	Interaction effect	0.61	3.19	0.621	0.29
Negative arousal^a^	Main effect	1.06	1.75	0.342	0.24
	Interaction effect	1.13	3.50	0.342	0.35
Reflection (theoretical contextualization)	Main effect	0.42	1	0.521	0.16
	Interaction effect	0.03	2	0.966	0.06
Reflection (theoretical evaluation)	Main effect	1.08	1	0.302	5.02
	Interaction effect	1.77	2	0.178	0.44

For emotional arousal there are three measuring times, but just two measuring times for reflection (posttest 1 and posttest 2). ^a^Correction according to Greenhouse–Geisser.

Concerning theoretical contextualization, there was no significant main effect [*F*(1,74) = 0.42, *p* = 0.521, *d* = 0.16] and no significant interaction of reflection and the group factor [*F*(2,74) = 0.03, *p* = 0.966, *d* = 0.06]. A similar model emerges concerning theoretical evaluation. There are no significant main [*F*(1,74) = 1.08, *p* = 0.302, *d* = 5.02] or interaction effects [*F*(2,74) = 1.77, *p* = 0.178, *d* = 0.44], but medium to high effect sizes. This is why the contrast comparison was calculated. There is a significant contrast comparison (*p* < 0.001) with a high effect size (*d* = 4.50) between ig2 and the other groups (ig1, and cg; see Table 3). In addition, mean values are higher in ig2 than in the other two groups (ig1, and cg; see Table 1). Therefore, hypothesis 2b can be partially accepted.

The correlation analysis (see Table 4) showed significant correlations of positive emotional arousal with both reflection scales (theoretical contextualization and evaluation) at both pre-and posttest. No significant correlations were found between negative emotional arousal and reflection. In addition, there were significant correlations between theoretical contextualization and theoretical evaluation, but not between positive and negative emotional arousal. In sum, hypothesis 3 may be partially accepted.

**Table 4:** Correlation analysis for emotional arousal and reflection.

	Posttest 1				Posttest 2			
	1	2	3	4	1	2	3	4
1. Positive arousal	1				1			
2. Negative arousal	−0.06				1						0.02	1		
3. Reflection (theoretical contextualization)	0.23*	−0.20			1				0.48**	−0.03	1	
4. Reflection (theoretical evaluation)	0.40**	−0.12	0.36**		1	0.35**	−0.02		0.42**	1
Means	3.03	1.34	2.46	2.85	2.84	1.34	2.48	2.82
Min	1.10	1.00	1.20	1.00	1.00	1.00	1.00	1.00
Max	4.70	3.00	3.60	4.00	4.50	3.40	3.80	4.00
SD	0.73	0.46	0.50	0.56	0.85	0.51	0.54	0.63
*N*	91	92	93	92	82	82	79	80

**Correlation is significant at the 0.01 level (2-tailed), *Correlation is significant at the 0.05 level (2-tailed).

### 5.4. Prediction of self-efficacy

Finally, it was assumed that emotional arousal as well as reflection predicts changes in self-efficacy. Five regression models were calculated to gradually include all independent variables in the model to learn the extent of the proportion of variance explained changes (see Table 5).

**Table 5:** Regression analysis: Predictors of self-efficacy (posttest 2).

	Model 1		Model 2		Model 3		Model 4		Model 5	
	*β*	*p*	*β*	*p*	*β*	*p*	*β*	*p*	*β*	*p*
Self-efficacy (pretest)	0.84	<0.001	0.79	<0.001	0.75	<0.001	0.76	<0.001	0.77	<0.001
Positive emotional arousal	0.04	0.345	−0.02	0.730	−0.03	0.555	−0.02	0.641	−0.02	0.680
Negative emotional arousal	−0.13	0.226	−0.14	0.187	−0.15	0.150	−0.15	0.167	−0.14	0.181
Reflection (theoretical contextualization)			0.22	0.016	0.23	0.012	0.24	0.010	0.24	0.011
Reflection (theoretical evaluation)			0.04	0.535	0.06	0.355	0.07	0.281	0.07	0.285
Prior experience in analyzing videos					0.13	0.124	0.13	0.112	0.13	0.123
Reflection skills							−0.04	0.303	−0.04	0.345
Interest and relevance									−0.01	0.864
Model fit	*R* = 0.77 *R*^2^ = 0.59 corrected *R*^2^ = 0.56		*R* = 0.81 *R*^2^ = 0.66 corrected *R*^2^ = 0.62		*R* = 0.82 *R*^2^ = 0.68 corrected *R*^2^ = 0.63		*R* = 0.83 *R*^2^ = 0.68 corrected *R*^2^ = 0.63		*R* = 0.83 *R*^2^ = 0.68 corrected *R*^2^ = 0.62	

Self-efficacy (posttest 2) as the dependent variable.

Model 1 shows that neither positive nor negative emotional arousal are significant predictors for changes in self-efficacy. However, reflection (theoretical contextualization) is a significant predictor (*p* = 0.016) for changes in self-efficacy (model 2). The added control variables of prior experience in analyzing video lessons (model 3), reflection skills (model 4), and interest and relevance (model 5) had no significant effect. Since the model quality does not change due to the addition of the control variables, it can be assumed that model 2 is the most informative as it explains most of the variance with only a few variables. While there are no effects of emotional arousal on self-efficacy, reflection is a significant predictor of self-efficacy. Therefore, hypothesis 4a must be rejected and hypothesis 4b may be accepted.

## 6. Discussion

The present study focused on the effects of emotion and reflection on student teachers’ changes in self-efficacy following video-based learning. The experimental study design includes two intervention groups in which student teachers analyzed video lessons and a control group in which participants analyzed text-based case studies. The observation tasks also varied: One intervention group and the control group received open-ended observation tasks, whereas the other intervention group received structured observation tasks for the video analysis. This variation aimed to evoke variance in students’ depth of reflection.

Our descriptive findings indicate that there are fairly high means for self-efficacy at all measurement points. Hence, even at the beginning of their teaching program, students assess their self-efficacy favorably concerning adaptive teaching in heterogeneous classrooms across all measurement points (upper range of the 4-point Likert scale). The results can also be partially attributed to the scale being limited to four rating options. Similar mean values are shown concerning the reflection scales. In all groups, the students rated their reflection concerning theoretical contextualization and theoretical evaluation as medium to high. In general, participants in all groups experience a higher positive emotional arousal compared to negative emotional arousal. This finding is in line with previous research studies, which also found more positive emotions than negative emotions in the context of video lesson analysis compared to a text-based control group (*cf.*, Syring et al., 2015). However, the means of positive emotional arousal remain relatively constant from Pretest to Posttest 2. This result is in contradiction with previous research. For example, Egloff and Souvignier (2020) showed increased positive emotions after video analysis. In the present study, only low negative emotional arousal was observed. This result goes against the findings of Kleinknecht and Poschinski (2014), who reported that analyzing the videos of others evoked negative emotions. One possible explanation is the conceptual differences between the studies. Specifically, Kleinknecht and Poschinski's (2014) study of teachers, who are fully trained and already teach, was conducted within the framework of qualitative design, whereas our study collected quantitative data from beginning student teachers. Thus, beginning students may not yet be able to perceive critical teaching situations sufficiently well because they lack the required knowledge and competencies. In addition, student teachers who are at the beginning of their studies may not have developed a sensitivity to the practical relevance of the situations described, and therefore may have a limited emotional reaction to the teaching sequences.

This study investigated changes in self-efficacy as a result of the video-based intervention. We found students’ self-efficacy increased, with a medium effect size, while analyzing video lessons with structured observation tasks. This result is in line with other studies, which reported that domain-specific self-efficacy increased through video-based interventions (Gold et al., 2017; Thiel et al., 2020). However, the findings of the present study did not show an increase for students who analyzed video lessons or text-based case studies with open-ended observation tasks. One possible explanation for these findings is that learners need to process information deeply on the situation presented and that simply presenting video lessons does not necessarily support deep learning processes. This assumption aligns with the findings that beginning students need to be well-guided to attain deeper levels of reflection (e.g., Blomberg et al., 2013; Gaudin and Chaliès, 2015). Therefore, it can be assumed that not only learning material (e.g., video lessons) by itself influences changes in self-efficacy, but also the instructional embedding of the videos (e.g., Kumschick et al., 2017). Thus, instructions should be designed in such a way that students are focused on the salient events in video lessons.

The second area of investigation focused on the role of emotions and reflection during the intervention. Contrary to our expectations, there were no significant differences in positive emotional arousal or negative emotional arousal between the intervention groups (analyzing video lessons) and the control group (analyzing text-based case studies). However, there were descriptive differences that did not achieve the level of significance. This descriptive result is in line with the findings of Syring et al. (2015), who reported higher emotional arousal for analyzing videos compared to analyzing texts. Furthermore, there was a significant and observable overall improvement in positive emotional arousal (without group differences), which is in line with the assumption, that positive emotions increase while analyzing case studies (*cf.*, Egloff and Souvignier, 2020). What could not be shown was the superiority of video-based case studies in this context. One possible explanation for the findings of the current study is that since students were at the beginning of their studies they were not able to make sufficient personal references to the presented classroom situations and thus attributed less meaning to them. Consequently, emotional arousal was lower (Lazarus and Lazarus, 1994). In addition, the medium effect sizes for results that are statistically non-significant indicate that the study should be replicated with a sample size based on a power analysis. For further research, it may be advantageous to use more stimulating videos (e.g., best vs. worst cases) to create more salient vicarious experiences and attribute more meaning to them (Blomberg et al., 2013). In summary, the role of emotional arousal in the analysis of video lessons needs further investigation. Which features of videos evoke emotional arousal and whether there are differences between novice and trained teachers remain unanswered.

In this study, we investigated the differences in depth of reflection between the intervention group with structured observation tasks, and two other groups with open-ended observation tasks (text-based and video). We assumed that students who analyzed the video lessons with structured observation tasks would show a higher depth of reflection. As expected, our results demonstrate partly that there are significant differences in reflection between the groups concerning open-ended observation tasks and the group concerning structured observation tasks. One possible explanation for this finding is that structured observation tasks lead to a deeper reflection compared to open-ended observation tasks. This assumption is supported by empirical work showing a greater depth of reflection among in-service teachers viewing their teaching (video) and completing structured observation tasks. Overall, these findings confirm the assumption that both the use of video lessons, particularly instructional embedding, are helpful for student learning (e.g., Blomberg et al., 2013; Syring et al., 2015). Our results indicate that analyzing video lessons with structured observation tasks leads to a deeper reflection of the instructional situation in the context of already learned theories.

Another area of investigation focused on the relationship between emotion and reflection. In this study, a significant relationship between reflection and positive arousal was found. There was no relationship between negative emotional arousal and reflection. This result goes against the findings of Kleinknecht and Poschinski (2014), who reported an association between negative emotions and depth of reflection in in-service teachers. However, in this study qualitative data from already trained teachers were used, whereas our study was based on quantitative data from beginning student teachers. One possible explanation for the findings of the current study is that beginning students have less previous experience. Consequently, they lack interpretation skills regarding negative situations, which are not negative enough in the context of typical classroom situations.

The final area of investigation focused on the role of emotions and reflection for changes in self-efficacy during video-based interventions. As expected, our results show that reflection has a significant effect on changes in self-efficacy. However, emotional arousal was not predictive of changes in self-efficacy. This means that students with deeper reflection showed a greater increase in self-efficacy. One possible explanation for the low emotional arousal in our study is that students have too little experience with teaching. Students at the beginning of their teacher education program see less personal connection in typical classroom situations. It can be assumed that these findings might change with different video material (e.g., Blomberg et al., 2013) or with more experienced students with higher interpretation skills (e.g., Lazarus and Lazarus, 1994; Santagata and Guarino, 2011).

### 6.1. Limitations of the study and future directions

This study has several limitations. First, the appropriate sample size (*N* = 107), which was calculated in advance using G*Power analyzes, could not be reached because 39% of students dropped out. We believe that with a larger sample, results with medium effects that did not show significant *p*-values in the current study would have been significant. Second, dropout analyzes showed no differences in study duration, prior experience, and self-efficacy, but there may be differences in students’ motivation. Many participants in the video group with structured observation tasks ended the intervention early. One possible explanation is that students probably worked more intensively and longer with the videos may be due to the more detailed observation tasks than students in the other groups (open-ended observation tasks), which is why they dropped out early. Future research studies could attempt more balance between groups and use incentives. Third, the data from self-report instruments may have been biased due to socially desirable responses as well as students´ lack of practical experience, which could have led to results that differ from those obtained using other methods such as behavioral observation. Fourth, the validity of the self-developed reflection scale was not verified. Fifth, only the area of theoretical reflection was observed. It seems worthwhile to evaluate different levels of reflection more objectively (e.g., analysis of the responses to the reflection tasks) and in more detail (e.g., Aeppli and Lötscher, 2016).

Finally, the selected video lessons did not polarize students strongly enough, which resulted in relatively low emotional arousal and little variance. To investigate the association of self-efficacy with emotional arousal more meaningfully, it might be more fruitful to use alternative video lessons such as worst and best practice examples (e.g., Blomberg et al., 2013). Future research could include more facets of reflection, motivation, and professional knowledge to generate deeper insights. Nonetheless, the results of our study provide valuable information about the relationship between emotion, reflection, and self-efficacy in video-based interventions and offer ideas for improving teacher education practices. Finally, future studies could include individual and contextual factors of the participants as control variables in the analyzes (e.g., Boler, 1997; Nussbaum, 2001; Feldman Barrett et al., 2007).

### 6.2. Conclusion

Although we were not able to confirm all of our hypotheses, this study contributes to a better understanding of video-based learning in teacher education. Only a few studies to date have explored the role of emotions and reflection in the development of self-efficacy in video-based learning environments. In conclusion, the findings of the present study indicate that beginning teaching students benefit from video lessons if the analysis is guided in a structured way. Nevertheless, the results could not show the superiority of the video format *per se*, as self-efficacy did not increase in the video group with open observation tasks. The results might be clearer if another control group had been examined with structured observation tasks. However, the results provide valuable support for the use of video lessons with structured observation tasks in initial teacher education.

In addition, the purpose of the study was to investigate the sources of self-efficacy. The results show that reflection is a significant predictor of self-efficacy, while the exact relationship with emotions is not yet entirely clear. These findings have the following implications: First, self-efficacy, emotional arousal, and reflection are associated with each other. Second, intervention with video lessons and structured observation tasks increased the self-efficacy of beginning students. Thus, future research needs to explore the emotional experience of student teachers in the context of video lesson analysis and the role of emotion and reflection in changes in self-efficacy.

## Data availability statement

The datasets presented in this article are not readily available because informed consent signed by participants stated that data were only accessible to the authors of this study. Requests to access the datasets should be directed to AS, anne.schlosser@uni-bamberg.de.

## Ethics statement

Ethical review and approval was not required for the study on human participants in accordance with the local legislation and institutional requirements. The patients/participants provided their written informed consent to participate in this study.

## Author contributions

AS and JP planned and conducted the study and drafted the manuscript. AS performed the statistical analyses. All authors discussed the results, contributed to the final manuscript, read and approved the submitted manuscript.

## Funding

This research was funded by “Stiftung Innovation in der Hochschullehre” as part of the “Digitale Kulturen der Lehre entwickeln (DiKuLe)” project. The funders had no role in the study design, data collection, analysis, decision to publish, or preparation of the manuscript. The authors are responsible for the content of this publication.

## Conflict of interest

The authors declare that the research was conducted in the absence of any commercial or financial relationships that could be construed as a potential conflict of interest.

## Publisher’s note

All claims expressed in this article are solely those of the authors and do not necessarily represent those of their affiliated organizations, or those of the publisher, the editors and the reviewers. Any product that may be evaluated in this article, or claim that may be made by its manufacturer, is not guaranteed or endorsed by the publisher.

## Footnotes

^1^University of Münster (2022) ProVision https://vsso.uni-muenster.de/ProVision/video/#MAT_K1_LK5_1ES_SEL [9:42 to 16:42, Accessed October 18, 2022].

^2^University of Münster (2022) ProVision https://vsso.uni-muenster.de/ProVision/video/#MAT_K1_LK4_3ES_LER [1:43to14:35;20:10 to 21: 57, Accessed October 18, 2022].

^3^University of Münster (2022) ProVision https://unterrichtsvideos.net/metaportal/ [Accessed October 18, 2022].
